# Impact of polyherbal formulation oral administration on the estrus response, luteal activity, and oxidative stress in postpartum dairy cows with ovarian subfunction

**DOI:** 10.14202/vetworld.2022.360-367

**Published:** 2022-02-17

**Authors:** Yahia A. Amin, Nasra Ahmed M. Youssef, Alaa-Eldin Zain-Elabdeen Mahmoud, Mohammed Salah, Atef M. H. Khalil, Obeid Shanab, Ahmed Saad Ahmed Hassaneen

**Affiliations:** 1 Department of Theriogenology, Faculty of Veterinary Medicine, Aswan University, Aswan, Egypt; 2 Department of Theriogenology, Obstetrics and Artificial Insemination, Faculty of Veterinary Medicine, South Valley University, Qena 83523, Egypt; 3 Department of Theriogenology, Faculty of Veterinary Medicine, Sohag University, Sohag, Egypt; 4 Department of Biochemistry, Faculty of Veterinary Medicine, South Valley University, Qena 83523, Egypt; 5 Department of Pathology and Clinical Pathology, Faculty of Veterinary Medicine, South Valley University, Qena 83523, Egypt; 6 Theriogenology Consultant, Qena Governmental Dairy Farms, Qena Governmental Animal Production Sector, Qena Governorate, Egypt

**Keywords:** estrous cycle, ovarian inactivity, phytotherapy, reproductive herbal medicine

## Abstract

**Background and Aim:**

The reproductive performance of dairy cows is of a high economic value to maintain efficient and sustainable productivity. Postpartum anestrus is one of the major infertility problems that cause limitation of dairy cow reproductive performance and productivity. The previous studies have reported using a polyherbal formulation for different purposes, including reproductive performance. This study was conducted to examine the efficacy of polyherbal formulation administration on the reproductive performance of cows and to establish the oral administration of polyherbal formulations as a safe, effective, and economic treatment for dairy cows with postpartum anestrus due to nutritional disorders, negative energy balance, high milk production, and/or heat stress.

**Materials and Methods:**

A total of 14 dairy cows with postpartum anestrus were randomly divided into two groups, which were subjected to oral administration of distilled water that served as the control (Ctrl; n=5) or polyherbal treatment (polyherbal treated; n=9) for two shots of treatment (each for 3 successive days) with a 10-day interval. Blood sampling and ultrasonography were performed before treatment and after the first and second shots of treatment. Progesterone (P4) assay was also performed.

**Results:**

The estrus induction rate was 66.7% after the first shot of treatment in the polyherbal-treated group, which increased significantly to 88.9% after the second shot of treatment, compared with the Ctrl group (20%). The estrus response was confirmed by ultrasonography and P4 hormone assay, wherein the polyherbal treatment significantly increased the P4 concentration in the polyherbal-treated group after the second shot of treatment compared to that before treatment and in the Ctrl group at all time points. On pregnancy diagnosis, the treated dairy cows showed conception rates of 66.7% and 20.0% in the polyherbal-treated and Ctrl groups, respectively. In contrast, the concentration of malondialdehyde, an oxidative stress marker, and the total antioxidant capacity remained unchanged between both groups before and after treatment.

**Conclusion:**

The polyherbal formulation containing tubers of *C. rotundus*, *M. pterygosperma*, rhizome of *Z. officinale*, and *A. cepa* has the potential to induce estrus response and luteal activity in dairy cows and is a possible treatment for ovarian inactivity in dairy farms.

## Introduction

Cows are the most important livestock for milk and meat production. They largely contribute to the agricultural economy worldwide [1]. The importance of cow production efficiency is increasing throughout the world to meet the increasing demands of human population for animal protein and milk. In several countries, including Egypt, beef meat is considered the major source of animal protein. Beef production in Egypt is insufficient for the increasing population with an estimated population of 105 million in 2022 [2]. Dairy cows normally calve for the 1^st^ time at 2 years of age with an average lifespan of 4.5-6 years for optimum production [3]. Several factors contribute to reduce the productivity of cows; reproduction is one of the primary factors affecting cow production, and any reproductive disorders have a negative impact on animal production [4]. Poor nutritional status of the cow after calving appears to alter both ovarian follicular development and luteal function, and it has been recently reported that there are 14 plasma differential proteins that are associated with inactive ovaries in dairy cows [5]. Anestrus is a broad term that indicates the lack of estrous signs despite efficient estrous detection [6]. In Egypt, anestrus and low conception rates are the major causes of economic losses and female culling in cattle herds, resulting in high annual losses [7]. Prolonged postpartum is a major limitation to high reproductive efficiency in cows, with suckling and nutrition being the major factors that influence the length of the postpartum period [8]. Different therapies, including hormonal therapy, have limitations, including high cost, withdrawal period, and adverse effects [9]. Non-hormonal polyherbal preparations are used to treat postpartum anestrus cows with ovarian subfunctions [9,10].

Polyherbal formulations have more potential and advantages over single herbal formulations because of the synergistic effects and several biological functions of their contents [11]. A recent study reported that the treatment of postpartum dairy buffaloes with herbal supplementations enhanced the resumption of ovarian cyclicity [12]. Several herbs are used as natural sources of minerals, vitamins, and antioxidants. For instance, *Cyperus rotundus* is rich in copper, magnesium, and manganese [10]; *Moringa pterygosperma* contains several vitamins and minerals, including calcium and phosphorus [13]; the rhizome of *Zingiber officinale* contains traces of iodine and fluorine [14]; and *Allium cepa* (onion) is a natural source of stigmasterol, cholesterol, β-sitosterol, and kaempferol [15,16]. Ashraf and Bhatti [17] recently reported that stigmasterol is one of the most important plant sterols due to its antioxidant and anti-inflammatory biological properties. Iodine supplementation has been reported to enhance folliculogenesis [18]. Furthermore, Galbat [19] recently reported that cows with ovarian inactivity exhibited deficiencies in trace elements such as copper and iodine. Studies have also reported that polyherbal formulations induced milk production in treated dairy cows [20,21] and rodents [22].

Moreover, polyherbal formulations were found to improve the somatic cell count and milk resistance profile in dairy cows with subclinical mastitis [23]. The efficacy of a polyherbal mixture in improving the milk yield resulted in the highest income with high economic value [20,21]. In addition, intrauterine infusion with polyherbal formulation was previously investigated in a field study to treat various reproductive infertility problems in cows [24]. It has been recently reported that grazing of small ruminants on pasture of different natural plants stimulated the enzymatic antioxidant defense in ewe in Mediterranean shrublands [25]. Another recently published study showed that supplementation of polyherbal formulation improved the antioxidant defense mechanism and blood biochemical indices in goats [26].

We hypothesized that oral administration of polyherbal formulations would be clinically beneficial in the treatment of ovarian inactivity in postpartum dairy cows. The present study aimed to evaluate the efficacy of polyherbal formulation administration on the reproductive performance of cows and to establish the oral administration of polyherbal formulations as a safe, effective, and economic treatment strategy for postpartum anestrus due to nutritional disorders, negative energy balance, high milk production, and/or heat stress.

## Materials and Methods

### Ethical approval

The study protocol was approved by the Animal Ethics and Use Committee of The Faculty of Veterinary Medicine, South Valley University Qena, Egypt (Approval no. FC, 236/2018).

### Study period and location

The field study was conducted from July 2019 to September 2019 and the laboratory analysis was conducted from August 2019 to February 2020. This study was conducted in Qena province, Egypt, located at 75 m above mean sea level, latitude 26.16° N, and longitude 32.72° E. The study was performed during the summer season (July-September) when the low (minimum) and high (maximum, °C) temperature ranged between 24-26°C and 39-41°C, respectively. The relative humidity ranged between 21% in July and 27% in September. The photoperiod throughout the whole period of the study extended from 13:60 h in July to 12:30 h in September.

**Figure-1:**
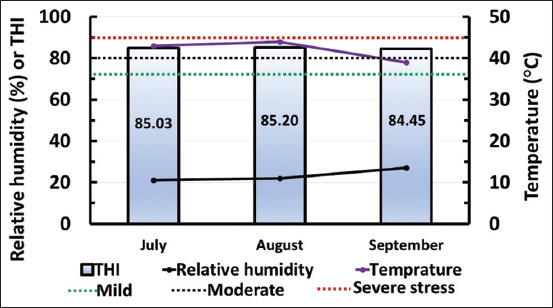
The temperature-humidity index (THI) and the weather data in the study location (Qena Province) during the experimental period of the study (July-September). The figure shows the weather data, including relative humidity (%; black line), maximum temperature (°C; violet line), and THI (gradient blue column). The threshold level for mild (72 THI), moderate (80 THI), and severe stress (90 THI) is shown as green, black, and red square dot lines, respectively. The values inside each column refer to the THI value during this month.

Weather data at Qena (July-September), including maximum temperature (T, °C) and relative humidity (RH, %) were used to determine the temperature-humidity index (THI) (Figure-1) using the following equation [27,28]:

THI = (1.8×T+32) – ([0.55–0.0055×RH) × (1.8×T–26)

The threshold value of heat stress in dairy cows was set as 72 points THI, where the stress was categorized into mild (72-79 THI), moderate (80-89 THI), and severe stress (≥90 THI) [29].

### Animals and experimental design

Of 326 Holstein dairy cows, 15 cows (aged 3-6 years; weighing 440-535 kg) suffering from postpartum anestrus for 3-5 months were used in this study. Dairy cows with a history of metritis, lameness, or any other disorders that may directly or indirectly affect the reproductive performance were excluded from the study. Cows were randomly divided into two groups; Polyherbal treated (n=9) and control (Ctrl; n=5). All dairy cows were housed in semi-open stalls and fed on a daily formulated ration of roughages and concentrates (1.5, and 1 kg per 100 kg body weight, respectively), with *ad libitum* access to water. The postpartum anestrus was due to ovarian inactivity that had been confirmed by rectal examination and ultrasonography. One of the 15 cows included in this study was excluded after the first shot of treatment because of the culling due to unresponsive mastitis.

### Oral administration of polyherbal formulation

For each cow in the polyherbal-treated group, 50 g of a polyherbal formulation (containing tubers of *C. rotundus*, *M. pterygosperma*, rhizome of *Z. officinale*, and *A. cepa*; 12.5 g of each herb) was dissolved in 150 mL distilled water immediately before being daily administered per os to postpartum anestrus cows.

**Figure-2:**
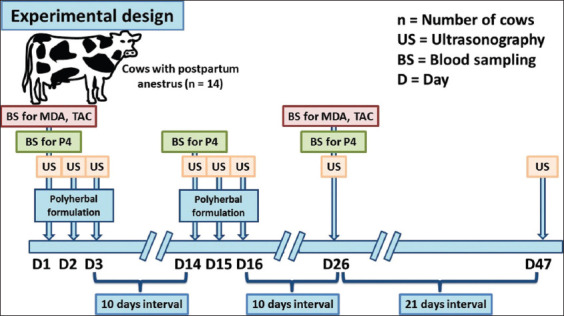
Schematic diagram showing the experimental protocol for the study. Postpartum dairy cows suffering from anestrum due to ovarian inactivity (n=14) were administrated either polyherbal formulation (n=9) or distilled water (Ctrl; n=5) for two shot treatments (each consists of 3 successive days with an interval of 10 days) as follows; day D1, D2, D3, D14, D15, and D16. Blood sampling was performed on D1, D14, and D26 for P4 assay, and on D1 (before treatment), and D26 (after treatment) for oxidative stress assays. Ultrasonography was performed on D1, D2, D3, D14, D15, D16, D26, and D47 [Source: This figure was prepared by all the authors in collaboration].

Either polyherbal formulation or distilled water was orally administered to the dairy cows for 3 successive days and repeated as a second shot for 3 other successive days after an interval of 10 days on day 1 (D1), D2, and D3 for the first shot of treatment and D14, D15, and D16 for the second shot of treatment (Figure-2).

### Blood sampling

Blood samples (10 mL) were collected from the coccygeal vein into plain tubes while performing transrectal ultrasonography, that was, D1, D14, and D26; where D1 was the start day of oral administration of the first shot of treatment (Figure-2). All blood samples were transferred to the laboratory in an ice box along with ice pack within 1 h, where sera were separated by centrifugation using laboratory centrifuge with fixed-angle rotor at 1000 × g for 15 min. Serum aliquots were stored at −20°C until hormonal analysis.

### Ultrasonography

Transrectal ultrasonography using ultrasound machine (SonoScape A5v Veterinary Ultrasound scanner; SonoScape Medical Corporation, China) with a multifrequency linear transducer (5-12 MHz) was performed on D1, D2, D3, D14, D15, D16, and D26 for evaluating the condition of ovaries and on D47 for pregnancy diagnosis (Figure-2).

### Progesterone (P4) hormone assay

Serum P4 concentrations were measured on D1, D14, and D26 for evaluating the luteal activity (Figure-2) by single enzyme-linked immunosorbent assay using P4 kit (ELITech clinical system, France). The absorbance was measured at a wavelength of 450 nm within 15 min.

### Oxidative stress assays

The Spectro Ultraviolet spectrophotometer (Labomed, Inc., Los Angeles, CA, USA) was used to determine the serum concentration of malondialdehyde (MDA) colorimetrically as an indicator of lipid peroxidation (Biodiagnostic commercial assay kits, Cairo, Egypt) and the serum levels of total antioxidant capacity (TAC) as an antioxidant biomarker kinetically (Biodiagnostic commercial assay kits). Both MDA and TAC levels were evaluated before the start of treatment (D1) to examine whether postpartum anestrus cows suffer from oxidative stress and after the treatment (D26) to examine whether polyherbal treatment could suppress the oxidative stress – if present in the polyherbal-treated cows (Figure-2).

### Statistical analysis

Data were arranged using Microsoft Excel 2010, and all values were expressed as mean±standard error of the mean. All data were analyzed using GraphPad Prism (GraphPad Software, San Diego, CA, USA) by one-way analysis of variance followed by a *post hoc* Tukey test. Results were considered to be statistically significant at p<0.05. Differences in estrus response and conception rate between the two groups were analyzed by Fisher’s exact test (p<0.05).

## Results

### Estrus response, conception rate, and ultrasonographic examination before treatment and after the first and second shot in the polyherbal-treated group

Cows in the polyherbal-treated group showed an estrus induction rate of 66.7% after the first shot of treatment and a highly significant estrus induction rate of 88.9% after the second shot of treatment. However, cows in the Ctrl group failed to show an estrus response after the first shot of treatment (using distilled water), and later, only 20% of the cows showed an estrus response after 14-16 days of the study (second shot of treatment by distilled water). The conception rates were 66.7% and 20.0% in the polyherbal-treated and Ctrl groups, respectively (Table-1).

**Table 1:** Estrus induction rate and conception rate in the Ctrl and polyherbal-treated postpartum anestrus dairy cows.

Treatment/number of animals	Ctrl group (%)	Polyherbal-treated group (%)
Estrus induction after the first shot	0/5 (00.0)	6/9 (66.7)
Estrus induction after the second shot	1/5 (20.0)	8/9 (88.9)*
Conception rate (%)	20.0	66.7

* Means bearing superscripts in the same row differ significantly (p<0.05). Ctrl=Control

**Figure-3:**
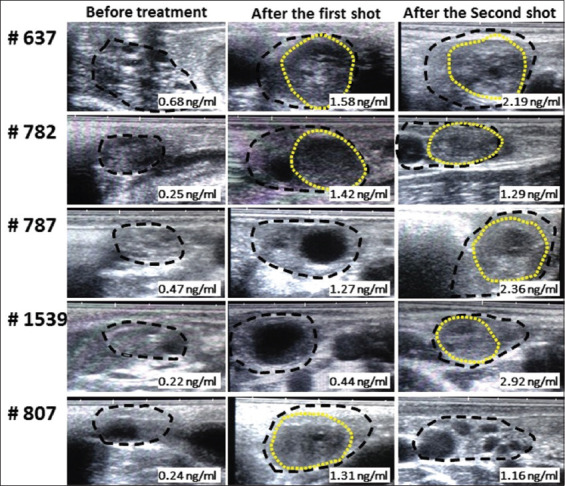
Representative ultrasonographic scan images of individual polyherbal-treated cows ovaries using transrectal ultrasonography before, after the first shot, and after the second shot treatment. The figure shows the whole ovaries (black dots outlines) with their ovarian structures either non-echogenic follicles and/or hyperechogenic corpora lutea (yellow dots outlines); before treatment (left panel), after the first shot of treatment (middle panel), and after the second shot of treatment (right panel). The individual cow’s progesterone concentrations (P4; ng/mL) at the time of ultrasonography are shown at the lower right corner of the ultrasonographic images.

Figure-3 shows the representative ultrasonographic images for individual cows treated with the polyherbal formulation before and after the first and second shot of treatment. The P4 concentrations of individual cows are indicated at the lower right corner of the ultrasonographic images at the corresponding time points (Figure-3).

### P4 concentrations (ng/mL) before treatment and after the first and second shot in Ctrl and polyherbal-treated groups

**Figure-4:**
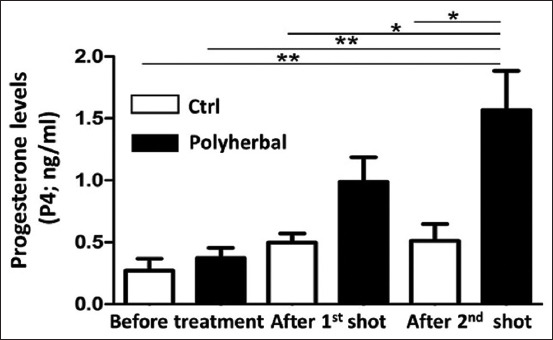
Progesterone concentrations (P4; ng/mL) before treatment, after the first shot treatment, and after the second shot in dairy cows suffering from anestrum due to ovarian inactivity (n=14) and subjected to oral administration of either polyherbal formulation (n=9) as black column or distilled water (Ctrl; n=5) as white column. Values presented as means±standard error of the mean, *p<0.05, **p<0.01 (one-way analysis of variance followed by post hoc Tukey’s test). Ctrl=Control.

Before treatment, the P4 concentration in both groups was <1 ng/mL, with the mean±standard error of the mean values being 0.27±0.10 and 0.37±0.08 ng/mL in the Ctrl and polyherbal-treated groups, respectively. Furthermore, no significant changes were observed between P4 concentrations in the two groups at this time point (Figure-4).

After the first shot of treatment, the P4 concentration in the polyherbal-treated group was slightly <1 ng/mL (0.99±0.20 ng/mL), whereas it was 0.51±0.14 ng/mL in the Ctrl group. Despite the higher concentration in the polyherbal-treated group, there were no significant changes between P4 concentrations in the two groups at this time point (Figure-4).

After the second shot of oral administration, the P4 concentration in the polyherbal-treated group was significantly higher than that before treatment and after the first shot of treatment (1.57±0.32 ng/mL). Moreover, the P4 concentration in the polyherbal-treated group after the second shot of treatment was significantly higher than that in the Ctrl group at all time points (Figure-4).

No significant changes were detected between P4 concentrations at all time points in the Ctrl group, with the values being 0.27±0.10, 0.50±0.07, and 0.51±0.14 ng/mL before treatment and after the first and second shot of treatment, respectively (Figure-4).

### MDA concentrations before and after the second shot of treatment in the Ctrl and polyherbal-treated groups

**Figure-5:**
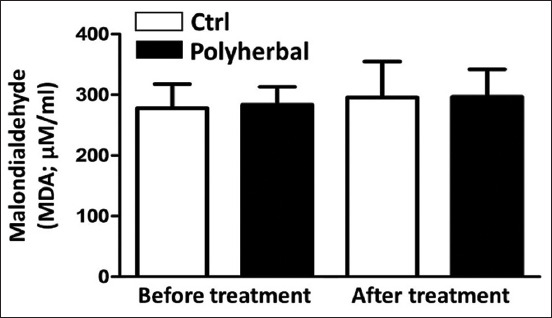
Malondialdehyde concentrations (MDA; μM/mL) before treatment and after the second shot of treatment in dairy cows suffering from anestrum due to ovarian inactivity (n=14) and subjected to oral administration of either polyherbal formulation (n=9) as black column or distilled water (Ctrl; n=5) as white column. Values presented as means±standard error of the mean, statistical analysis was performed using one-way analysis of variance followed by post hoc Tukey’s test. Ctrl=Control.

No significant changes were detected in the MDA concentration before and after the second shot of treatment (Figure-5). Furthermore, the MDA concentration in the polyherbal-treated group after the second shot of treatment was not significantly different from the before treatment (Figure-5).

### TAC levels before and after the second shot of treatment in the Ctrl and polyherbal-treated groups

**Figure-6:**
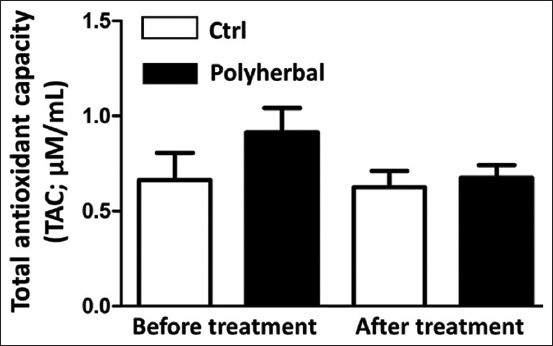
Total antioxidants capacity concentrations (TAC; μM/mL) before treatment and after the second shot of treatment in dairy cows suffering from anestrum due to ovarian inactivity (n=14) and subjected to oral administration of either polyherbal formulation (n=9) as black column or distilled water (Ctrl; n=5) as white column. Values presented as means±standard error of the mean, statistical analysis was performed using oneway analysis of variance followed by *post hoc* Tukey‘s test. Ctrl=Control.

The TAC levels showed no significant changes between both groups at all time points (before and after treatment). The TAC levels before treatment were 0.66±0.14 and 1.06±0.19 μM/mL in the Ctrl and polyherbal-treated groups, respectively, which changed after treatment to 0.63±0.09 and 0.68±0.07 μM/mL, respectively (Figure-6). Furthermore, the TAC levels in the polyherbal-treated group after the second shot of treatment were not significantly different from those before treatment (Figure-6).

## Discussion

This study has described the reproductive and antioxidant response of anestrus dairy cows with inactive ovaries to the oral administration of a polyherbal formulation during the summer season. Different therapies for anestrus due to ovarian inactivity have been previously examined in postpartum dairy cows and buffaloes [30-35]. Nevertheless, there exists a need to improve the efficacy, side effects related hazards, and economic impact of these therapies.

The present study demonstrated a significant improvement in the estrus induction rate and increased conception rate in the polyherbal-treated cows. These findings are consistent with those of an *in vivo* experimental study conducted in goats, which also demonstrated an increase in conception rate in the polyherbal-treated group (62.5%) compared with the Ctrl group (33.3%) [36]. Another recent study also reported that polyherbal mixture administration improved the postpartum reproductive performance of Sahiwal cows, including the estrus response, number of services per conception, and the first service conception rate [37].

The high concentration of P4 of >1 ng/mL in response to the oral administration of polyherbal formulation confirmed the luteal activity and is consistent with the other findings of estrus response and ultrasonographic examinations in this study. Such high P4 concentration is an evidence of ovulation, indicating that the ovulated follicle was transformed into a P4-producing corpus luteum. High P4 levels are responsible for the subsequent increase in the responsiveness to E2 [38]. The resumption of the ovarian activity in polyherbal-treated anestrus cows observed in this study is probably attributed to enhanced E2 synthesis due to the high cholesterol and phytosterol (stigmasterol and β-sitosterol) contents in *A. cepa* [15,16,39]. Keen *et al*. [40] reported that manganese contributes as a cofactor in cholesterol synthesis, which is essential for the synthesis of steroids, including E2. High E2 levels positively feedback to the hypothalamus, induce GnRH secretion and subsequently induce ovulation [41]. Furthermore, Wang *et al*. [42] reported that kaempferol, which is present in *A. cepa*, is a selective estrogen receptor (ER) modulator that enhances the activities of both ERa and ERb. Oral administration of a polyherbal formulation significantly improved the secretion of reproductive hormones, namely, luteinizing hormone (LH), follicle-stimulating hormone (FSH), and sex steroid hormones, and also improved the spermatogenic activity in oligospermic infertile men [43]. From the viewpoint of nutrition, it is necessary to maintain the normal levels of macroelements and microelements for reproductive function in dairy cattle [44,45]. Such maintenance was probably achieved by the addition of the essential macroelements such as calcium and phosphorus present in *M. pterygosperma* [13], microelements (trace elements) such as copper and manganese present in *C. rotundus* [10], and iodine present in the rhizome of *Z. officinale* [14]. The improvement in the ovarian activity observed in the present study is consistent with a previous study that showed that polyherbal formulations significantly increased FSH and LH secretion in female rats and a significant increase in ovarian activity [46].

According to the weather data and THI reported during the study period (July-September), dairy cows suffered from moderate stress. In addition, there was no significant difference in the levels of MDA and TAC before and after treatment. Although the previous studies have reported that a polyherbal formulation containing onion skin extract exhibited antioxidant properties [15,16], the balance between oxidative stress and antioxidant response would be due to the efficiency of the cooling system in the farm, which minimizes the effect of heat stress. Another reason reported previously was that the supplementation of a polyherbal formulation enhanced the P4 profile in female goats more likely by suppressing the production of reactive oxygen species [26]. This explanation is also supported by a previous study that examined the effect of CIDR treatment on oxidant/antioxidant biomarkers in summer-stressed anestrous buffaloes, in which the TAC levels remained low despite the increase in P4 levels after the resumption of ovarian activity [31]. Recent studies have reported that the supplementation of phytogenics as feed additives has beneficial effects on the reproductive performance of ruminant animals, including the effect on sexual behavior and hormonal profiles, and those findings were based on both *in vivo* and *in vitro* experiments [47,48].

## Conclusion

Our study indicated that oral administration of the polyherbal formulation (containing tubers of *C. rotundus, M. pterygosperma*, rhizome of *Z. officinale*, and *A. cepa*) was beneficial in improving the reproductive performance of dairy cows that administration of polyherbal formulation treated the ovarian inactivity in dairy cows as it had the potential to resume ovarian activity, induce estrus and luteal activity, increase P4 concentrations, and finally increase the conception rate in dairy cows. However, a limitation was that our study was conducted on a group of postpartum anestrus dairy cows in one herd, and another limitation was that the levels of macroelements and microelements were not determined before and after treatment. Therefore, for clinical application, further studies are required on large numbers and different herds with multiple doses and concentrations of polyherbal supplementations along with the evaluation of the levels of the related essential minerals before and after treatment. This would help in elucidating the most appropriate and economic use of polyherbal formulation to generalize its clinical use in the treatment of ovarian inactivity and improvement of reproductive performance in dairy cows. Further studies on the effect polyherbal formulation’s administration on milk production could be more economical and beneficial in improving both the milk production and reproductive performance of dairy cows.

## Authors’ Contributions

AEZEM, ASAH, and YAA: Conception and design of the study. ASAH, AEZEM, and YAA: Conducted the field study, examination of dairy cows, blood sampling, and ultrasonographic examination. ASAH, AMHK, MS, OS, and NAMY: Collected laboratory samples and conducted biochemical analyses. ASAH, NAMY, and MS: Manipulated and analyzed the data. ASAH, YAA, MS, NAMY, and OS: Performed data curation and interpretation of data. ASAH, YAA, and NAMY: Drafted the manuscript. AEZEM, ASAH, YAA, NAMY, AMHK, MS, and OS: Carried out final writing, critical review, and revision. All authors have read and approved the final manuscript.
